# Differences in receipt of recommended eye examinations by comorbidity status and healthcare utilization among nonelderly adults with diabetes

**DOI:** 10.1111/1753-0407.13328

**Published:** 2022-10-26

**Authors:** Cindy X. Cai, Minchul Kim, Elizabeth A. Lundeen, Stephen R. Benoit

**Affiliations:** ^1^ Wilmer Eye Institute, Johns Hopkins Hospital Baltimore Maryland USA; ^2^ Center for Outcomes Research, Department of Internal Medicine University of Illinois College of Medicine Peoria Peoria Illinois USA; ^3^ Division of Diabetes Translation National Center for Chronic Disease Prevention and Health Promotion, Centers for Disease Control and Prevention Atlanta Georgia USA

**Keywords:** diabetes mellitus, eye diseases, multiple chronic conditions, vision screening, 糖尿病, 多种慢性病, 视力筛查, 眼科疾病

## Abstract

**Background:**

To evaluate the effect of diabetes comorbidities by baseline healthcare utilization on receipt of recommended eye examinations.

**Methods:**

Retrospective analysis of 310 691 nonelderly adults with type 2 diabetes in the IBM MarketScan Commercial Database from 2016 to 2019. Patients were grouped based on diabetes‐concordant (related) or ‐discordant (unrelated) comorbidities. Logistic regression was used to estimate the prevalence ratio (PR) for eye examinations by comorbidity status, healthcare utilization, and an interaction between comorbidities and utilization, controlling for age, sex, region, and major eye disease.

**Results:**

Prevalence of biennial eye examinations varied by the four comorbidity groups: 43.5% (diabetes only), 52.7% (concordant + discordant comorbidities), 48.0% (concordant comorbidities only), and 45.3% (discordant comorbidities only). In the lowest healthcare utilization tertile, the concordant‐only and concordant + discordant groups had lower prevalence of examinations compared to diabetes only (PR 0.95 [95% CI 0.92–0.98] and PR 0.91 [95% CI 0.88–0.95], respectively). In the medium utilization tertile, the discordant‐only and concordant + discordant groups had lower prevalence of examinations (PR 0.89 [0.83–0.95] and PR 0.94 [0.90–0.98], respectively). In the highest utilization tertile, the concordant‐only and concordant + discordant groups had higher prevalence of examinations.

**Conclusions:**

Among patients with low healthcare utilization, having comorbid conditions is associated with lower prevalence of eye examinations. Among those with medium healthcare utilization, patients with diabetes‐discordant comorbidities are particularly vulnerable. This study highlights populations of diabetes patients who would benefit from increased assistance in receiving vision‐preserving eye examinations.

## Abstract 2

Highlights

Patients with diabetes who have comorbid medical conditions and are infrequent healthcare utilizers are at increased risk for not receiving recommended eye examinations.

## 摘要

**背景:**

通过基础医疗利用评估糖尿病合并症对接受推荐眼科检查的影响。

**方法:**

对IBM® MarketScan® 商业数据库2016‐2019年的310691例非老年2型糖尿病患者进行回顾性分析。根据糖尿病相关或无关合并症对患者进行分组。在控制年龄, 性别, 地区和主要眼病的情况下，采用Logistic回归方法估计眼科检查的患病率(PR)，通过合并症状况, 医疗保健利用度, 以及合并症和利用度之间的相互作用来评估。

**结果:**

两年一次的眼科检查的患病率在四个合并症组中有所不同:43.5%(单纯糖尿病组), 52.7%(相关和无关合并症并存组), 48.0%(仅相关合并症组)和45.3%(仅无关合并症组)。在卫生保健利用率最低三分位数中，仅相关组和并存组的检查率低于仅糖尿病组(PR分别为0.95[95%CI 0.92~0.98]和PR 0.91[0.88~0.95])。在中等利用率组中，仅无关组和并存组的检查率较低(PR分别为0.89[0.83~0.95]和0.94[0.90~0.98])。在利用率最高的三分位数中，仅相关组和并存组的检查患病率较高。

**结论:**

在医疗保健利用率低的患者中，合并疾病与较低的眼科检查患病率相关。在那些医疗保健利用率中等的人中，患有无关共病的糖尿病患者特别容易受到伤害。这项研究强调了糖尿病患者人群将受益于接受保护视力的眼科检查

## 1 INTRODUCTION

Diabetes is the leading cause of incident blindness among working‐age adults in the United States.^1^, ^2^ Vision loss can be prevented but requires regular eye examinations for early detection and prompt treatment of vision‐threatening complications.^3^, ^4^, ^5^ Eye care utilization can be adversely impacted by patients' comorbid medical conditions (e.g., hypertension, hyperlipidemia). Research has shown that 97.5% patients with diabetes mellitus have at least one comorbid condition and 88.5% have at least two.^6^ Competing demands from the comorbid conditions combined with time or resource constraints can limit effective diabetes care.^7^, ^8^ Discordant comorbidities, conditions that are not related to the pathogenesis or management plans for diabetes (e.g., arthritis, depression), may be more likely to negatively impact care than concordant comorbidities, conditions that share a pathophysiologic profile and clinical treatment plan similar to diabetes (e.g., hypertension, hyperlipidemia).^9^ Indeed, studies examining glycemic control among individuals with diabetes have found lower odds of meeting the glycosylated hemoglobin goal in those with discordant comorbidities compared to concordant ones.^10^

The impact of comorbid medical conditions on receipt of eye examinations is unclear.^7^, ^11^, ^12^, ^13^, ^14^ Eye care utilization is particularly sensitive to the competing demands from comorbid medical conditions because it typically requires a separate office visit to an optometrist or ophthalmologist. Furthermore, successful preventive care for diabetes also depends on the frequency of healthcare utilization, whereby more frequent users of the healthcare system are more likely to receive preventive care.^15^ The present study examines the effect of concordant + discordant comorbidities on receipt of recommended eye examinations for patients with type 2 diabetes and whether that effect varies by baseline healthcare utilization.

## 2 METHODS

### 2.1 Data source

The IBM MarketScan Commercial Database was used to identify a longitudinal, observational cohort of patients with type 2 diabetes mellitus.^16^ These data included deidentified patient‐level health insurance claims across the continuum of care (e.g., inpatient, outpatient, outpatient pharmacy) as well as enrollment data on working‐age adults <65 years of age and their dependents whose employers or health plans contributed data to the MarketScan database. This research was considered exempt from institutional review board review under 45 Code of Federal Regulations 46.101[b] [5], which covers Department of Health and Human Services research and demonstration projects which are designed to study, evaluate, or examine public benefit or service programs.

### 2.2 Patient selection

Adults aged 20 to <65 years with noncapitated insurance plans and continuous insurance enrollment in medical and pharmaceutical benefits from January 1, 2016 to December 31, 2019 were included in the analysis. The *International Classification of Diseases, Tenth Revision, Clinical Modification* (ICD‐10‐CM) diagnosis codes associated with inpatient and outpatient encounters during the baseline period from January 1, 2016 to December 31, 2017 were extracted. During this 2‐year period, patients were defined as having diabetes mellitus if they had at least one inpatient diabetes ICD‐10‐CM diagnosis code or any combination of two instances of the following: (A) an outpatient diabetes ICD‐10‐CM diagnosis code, (B) a filled prescription for insulin, sulfonylureas, meglitinides, sodium glucose cotransporter 2 inhibitors, thiazolidinediones, glucagon‐like peptide 1 (GLP‐1) receptor agonists, metformin, or other antidiabetic medications (Table S1, Figure S1). Two instances of the same type (e.g., two outpatient codes) qualified only if the events occurred on separate days. Two diabetes medication prescriptions also met the definition; however, prescriptions for metformin, thiazolidinediones, or GLP‐1 agonists had to be combined with another event type to qualify.

Patients with ≥3 diabetes diagnosis codes could be differentiated for type 1 or type 2 diabetes (Figure S1). Patients with <3 diabetes diagnosis codes were excluded from the analysis as well as those with type 1 diabetes mellitus. Type 1 was defined as: (1) >50% of the diabetes diagnosis codes were for type 1 diabetes, and (2) there was no prescription for a sulfonylurea^17^ (Table S1). Patients were defined as having type 2 diabetes mellitus if (1) >50% of the diabetes diagnosis codes were specific to type 2 diabetes, or (2) they did not meet the definition for type 1 diabetes and had a prescription for a noninsulin diabetes medication^17^ (Table S1). Patients with type 1 diabetes were excluded because they have different diabetic retinopathy screening guidelines than patients with type 2. For patients with type 2 diabetes, a diabetic retinopathy examination is recommended at the time of the diabetes diagnosis, and for patients with type 1 diabetes, it is recommended within 5 years after the onset of diabetes.^3^, ^4^ Without knowing the date of incident diabetes, we were unable to confirm that a patient with type 1 diabetes mellitus met the recommended criteria for a diabetic retinopathy examination.

### 2.3 Outcome

The outcome of interest was receipt of recommended biennial or annual diabetic eye examinations.^3^, ^4^ Eye examinations were defined based on the presence of qualifying administrative codes^17^, ^18^ (Table S1). The primary outcome was receipt of recommended biennial diabetic eye examinations, defined as the presence of at least one eye examination in the January 1, 2018 to December 31, 2019 period. The secondary outcome used for sensitivity analysis was receipt of annual diabetic eye examinations, defined as the presence of at least two eye exams separated by at least 10 months during the same time period.

### 2.4 Baseline covariates

Data from January 1, 2016 to December 31, 2017 were used to establish the baseline covariates for each patient.

#### 2.4.1 Comorbidity status

The main explanatory variable was comorbidity status. Medical comorbidities were defined based on the presence of ≥1 qualifying ICD‐10‐CM codes associated with inpatient and outpatient encounters.^19^, ^20^ Comorbidities were then categorized as diabetes concordant (hypertension, hyperlipidemia, congestive heart failure, coronary artery disease, cardiac arrhythmia, stroke, chronic kidney disease) or discordant (arthritis, osteoporosis, cancer, asthma, chronic obstructive pulmonary disease, dementia, depression, autism spectrum disorder, schizophrenia, hepatitis, human immunodeficiency virus, substance abuse disorder)^7^ (Table S2). Patients were placed into mutually exclusive groups: diabetes only (without any concordant or discordant comorbidities), concordant‐only comorbidities, discordant‐only comorbidities, or concordant + discordant comorbidities.^13^

#### 2.4.2 Other baseline covariates

A patient's history of major eye disease was represented as a binary variable which indicated the presence of age‐related macular degeneration or glaucoma^21^, ^22^ (Table S1). Other covariates including the patient's sex and geographic region (Northeast, North Central, South, West) were extracted from the earliest encounter in the baseline period. The patient's age is reported as the age at the end of the baseline period (December 31, 2017).

#### 2.4.3 Healthcare utilization

We enumerated the total number of outpatient encounters, not including emergency department or hospital admissions, during the baseline period and grouped patients into tertiles of low, medium, and high healthcare utilizers^23^ (Table S1).

### 2.5 Statistical analysis

Student's *t* test and analysis of variance were used to test differences between continuous variables, and the Pearson's chi‐squared test was used for categorical variables. Logistic regression analysis was used to estimate the prevalence ratio (PR) for receipt of diabetic eye examinations by baseline comorbidity status (reference: diabetes‐only group), healthcare utilization, and an interaction term between comorbidity status and utilization, while controlling for age, sex, region, and major eye disease.^24^ The statistical significance of the interaction term was assessed using the Wald test. Statistical significance was set at *p* < 0.05. All statistical analyses were performed in SAS/STAT software (Version 9.4 of the SAS system for Windows; SAS Institute Inc., Cary, North Carolina) and Stata (StataCorp 2019, Stata Statistical Software: Release 16; StataCorp LLC, College Station, Texas).

## 3 RESULTS

Of the 7 896 603 enrollees with continuous medical and pharmaceutical coverage from January 1, 2016 to December 31, 2019 in the MarketScan databases, a total of 310 691 patients with type 2 diabetes were included in the analysis (Table 1). Almost half (46.7%) were age 50 to <60 years and 54.1% were male. The majority (51.8%) were from the South, while 18.9% were from the Northeast, 19.1% from North Central, and 10.2% from the West. A small proportion (3.2%) had major eye disease. Healthcare utilization varied by comorbidity status, with the highest utilization of outpatient encounters in the concordant + discordant group (median 27 outpatient visits in 2 years, interquartile range [IQR] 16–44), followed by the discordant‐only group (median 19, IQR 12–33) and the concordant‐only group (median 14, IQR 8–22), and it was the lowest in the diabetes‐only group (median 9, IQR 5–16; *p* < 0.001; data not shown).

**TABLE 1:** Baseline demographic characteristics of participants by comorbidity status

	Total *N* = 310 691 (%)	Diabetes only *n* = 6858 (%)	Concordant only *n* = 141 627 (%)	Discordant only *n* = 4667 (%)	Concordant and discordant *n* = 157 539 (%)	*p* value
Age						<0.001
20 to <40 years	11 973 (3.9)	879 (12.8)	5879 (4.2)	519 (11.1)	4696 (3.0)	
40 to <50 years	53 013 (17.1)	1906 (27.8)	26 905 (19.0)	1200 (25.7)	23 002 (14.6)	
50 to <60 years	144 986 (46.7)	2833 (41.3)	66 798 (47.2)	1987 (42.6)	73 368 (46.6)	
60 to <65 years	100 719 (32.4)	1240 (18.1)	42 045 (29.7)	961 (20.6)	56 473 (35.9)	
Sex						<0.001
Female	142 606 (45.9)	3296 (48.1)	53 770 (38.0)	2983 (63.9)	82 577 (52.4)	
Male	168 085 (54.1)	3562 (51.9)	87 857 (62.0)	1684 (36.1)	74 982 (47.6)	
Region						<0.001
Northeast	58 782 (18.9)	1124 (16.4)	25 517 (18.0)	967 (20.7)	31 174 (19.8)	
North Central	59 199 (19.1)	1454 (21.2)	25 959 (18.3)	1031 (22.1)	30 755 (19.5)	
South	161 065 (51.8)	2973 (43.4)	75 142 (53.1)	1904 (40.8)	81 046 (51.5)	
West	31 645 (10.2)	1307 (19.1)	15 009 (10.6)	765 (16.4)	14 564 (9.2)	
Major eye disease						<0.001
No	300 895 (96.9)	5571 (81.2)	137 468 (97.1)	2932 (62.8)	87 064 (55.3)	
Yes	9796 (3.2)	1287 (18.8)	4159 (2.9)	1735 (37.2)	70 475 (44.7)	
Healthcare utilization^a^						<0.001
Low (0–13)	105 159 (33.9)	4614 (67.3)	70 728 (49.9)	1515 (32.5)	28 302 (18.0)	
Medium (14–27)	103 849 (33.4)	1608 (23.5)	47 658 (33.7)	1607 (34.4)	52 976 (33.6)	
High (28–731)	101 683 (32.7)	636 (9.3)	23 241 (16.4)	1545 (33.1)	76 261 (48.4)	

^a^ Number of outpatient encounters in the baseline 2‐year period.

Prevalence of biennial eye examinations varied by comorbidity status. The majority (52.7%) in the concordant + discordant group received exams, while the prevalence of receiving an exam was 48.0% in the concordant‐only group, 45.3% in the discordant‐only group, and 43.5% in the diabetes‐only group (*p* < 0.001) (Table 2). In unadjusted logistic regression analysis, compared to the diabetes‐only group, both the concordant‐only and concordant + discordant groups had a higher PR for receiving exams (1.10, 95% CI 1.07–1.13, *p* < 0.001, and 1.21, 95% CI 1.18–1.24, *p* < 0.001, respectively), while the discordant‐only group was not statistically different from the diabetes‐only group (Table 2). Similar results were noted in the sensitivity analysis using the secondary outcome, where 23.6% in the concordant + discordant group received annual diabetic eye examinations, 20.9% in the concordant‐only group, 16.6% in the discordant‐only group, and 15.9% in the diabetes‐only group (*p* < 0.001) (Table S3).

**TABLE 2:** Adjusted and unadjusted prevalence ratio of receiving recommended biennial eye examination

	Received recommend eye exam n (%)	Unadjusted prevalence ratio (95% CI)	*p* value	Adjusted prevalence ratio (95% CI)	*p* value
Comorbidity status				(See Table 3)	
Diabetes only	2982 (43.5)	(Reference)			
Concordant only	67 919 (48.0)	1.10 (1.07, 1.13)	<0.001		
Discordant only	2112 (45.3)	1.04 (1.00, 1.08)	0.059		
Concordant and discordant	82 938 (52.7)	1.21 (1.18, 1.24)	<0.001		
Healthcare utilization^a^				(See Table 3)	
Low (0–13)	41 795 (39.7)	(Reference)			
Medium (14–27)	53 667 (51.7)	1.30 (1.29, 1.31)	<0.001		
High (28–731)	60 489 (59.5)	1.50 (1.48, 1.51)	<0.001		
Age					
20 to <40 years	4095 (34.2)	(Reference)		(Reference)	
40 to <50 years	21 948 (41.4)	1.21 (1.18, 1.24)	<0.001	1.14 (1.12, 1.16)	<0.001
50 to <60 years	71 516 (49.3)	1.44 (1.41, 1.48)	<0.001	1.29 (1.27, 1.31)	<0.001
60 to <65 years	58 392 (58.0)	1.70 (1.65, 1.74)	<0.001	1.45 (1.43, 1.47)	<0.001
Sex					
Female	74 574 (52.3)	(Reference)		(Reference)	
Male	81 377 (48.4)	0.93 (0.92, 0.93)	<0.001	0.96 (0.95, 0.97)	<0.001
Region					
Northeast	34 249 (58.3)	(Reference)		(Reference)	
North Central	30 366 (51.3)	0.88 (0.87, 0.89)	<0.001	0.91 (0.90, 0.92)	<0.001
South	76 577 (47.5)	0.82 (0.81, 0.82)	<0.001	0.85 (0.84, 0.85)	<0.001
West	14 759 (46.6)	0.80 (0.79, 0.81)	<0.001	0.85 (0.84, 0.86)	<0.001
Major eye disease					
No	147 144 (48.9)	(Reference)		(Reference)	
Yes	8807 (89.9)	1.84 (1.82, 1.85)	<0.001	1.78 (1.76, 1.79)	<0.001

^a^ Number of outpatient encounters in the baseline 2‐year period.

In the multivariable analysis, there was a statistically significant effect measure modification on comorbidity status by healthcare utilization (*p* < 0.001). In the lowest tertile of utilization (0–13 outpatient encounters in the baseline 2‐year period), the concordant‐only group had a 5% lower prevalence of biennial eye examinations compared to the diabetes‐only group (PR 0.95, 95% CI 0.92–0.98, *p* = 0.001), and the concordant + discordant group had a 9% lower prevalence (PR 0.91, 95% CI 0.88–0.95, *p* < 0.001) (Table 3). The discordant‐only group had a 5% lower prevalence compared to the diabetes‐only group, but this was not statistically significant (PR 0.95, 95% CI 0.89–1.02, *p* = 0.136). In the sensitivity analysis using the secondary outcome of annual eye exams, the discordant‐only and concordant + discordant groups had a lower PR compared to the diabetes‐only group (Table S3). In the medium tertile of utilization (14–27 outpatient encounters in the baseline 2‐year period), the discordant‐only group had an 11% lower prevalence of eye examinations compared to the diabetes‐only group (PR 0.89, 95% CI 0.83–0.95, *p* = 0.001), and the concordant + discordant group had a 6% lower prevalence (PR 0.94, 95% CI 0.90–0.98, *p* = 0.007) (Table 3). This was qualitatively similar in the sensitivity analysis (Table S3). Finally, in the highest tertile of utilization (28–731 outpatient encounters in the 2‐year period), both the concordant‐only and concordant + discordant groups had higher prevalence of eye examinations compared to diabetes only (PR 1.19, 95% CI 1.10–1.29, *p* < 0.001, and PR 1.15, 95% CI 1.07–1.25, *p* < 0.001, respectively), and the discordant‐only group was similar to the diabetes‐only group (Table 3).

**TABLE 3:** Adjusted prevalence ratio of receiving recommended biennial eye examinations by comorbidity status and stratified by baseline healthcare utilization

	Low utilization (0–13)^a^			Medium utilization (14–27)			High utilization (28–731)		
Diabetes only	(Reference)	95% CI	*p* value	(Reference)	95% CI	*p* value	(Reference)	95% CI	*p* value
Concordant only	0.95	0.92, 0.98	0.001	1.01	0.97, 1.06	0.586	1.19	1.10, 1.29	<0.001
Discordant only	0.95	0.89, 1.02	0.136	0.89	0.83, 0.95	0.001	1.06	0.97, 1.16	0.211
Concordant and discordant	0.91	0.88, 0.95	<0.001	0.94	0.90, 0.98	0.007	1.15	1.07, 1.25	<0.001

^a^ Number of outpatient encounters in the baseline 2‐year period.

Age influenced receipt of diabetic eye examinations. All age groups above 40 years had a higher prevalence of receiving diabetic eye examinations compared to those 20 to <40 years (Table 2). Males had a 4% lower prevalence of examinations compared to females. Patients living in the North Central, South, and West had lower prevalence of exams compared to those living in the Northeast. Finally, those with a major eye disease had a 78% higher prevalence of eye examinations compared to those without.

## 4 DISCUSSION

Using the MarketScan database, we found that receipt of recommended eye examinations varied by comorbidity status and frequency of outpatient healthcare utilization. Among low healthcare utilizers, having any comorbidity in general decreased the prevalence of eye examinations, although this decrease only achieved statistical significance in the concordant‐only and concordant + discordant groups. Among medium healthcare utilizers, individuals with discordant comorbidities, whether discordant only or concordant + discordant, were less likely to receive recommended eye examinations. Among the high healthcare utilizers, having comorbidities no longer negatively impacted receipt of eye examinations. This study highlights specific populations of patients with diabetes that can be identified on the basis of their comorbid medical conditions and healthcare utilization who would benefit from increased assistance in receiving vision‐preserving eye examinations.

In the present study, among low healthcare utilizers, having comorbidities, whether concordant or concordant + discordant, was associated with decreased receipt of eye examinations. From the patient's perspective, having to manage multiple comorbid conditions can present a number of barriers to self‐care including financial constraints, physical limitations caused by the medical conditions, total burden of medications, and other logistical issues.^25^ Adequately addressing multiple comorbid conditions can also be challenging from the provider's perspective. It may be difficult to deliver high‐quality diabetes care over a smaller number of office encounters given the complexity of diabetes management.^9^, ^15^ There are competing demands on the physicians who must address patient concerns, chronic illness care, or prevention and counseling services.^26^ Indeed, studies have demonstrated a relationship between the frequency of outpatient encounters with receipt of American Diabetes Association (ADA) recommendations for semiannual glycosylated hemoglobin monitoring, annual retinal examinations, and annual microalbuminuria testing.^15^ Specifically, patients with fewer than eight visits during a 2‐year study period or visits for lower‐priority conditions had reduced odds of receiving the ADA‐recommended diabetes preventive care.

The differential impact of the type of comorbid condition on receipt of diabetic eye examination was most obvious among the medium healthcare utilizers. In this group, the findings support our hypothesis that discordant comorbidities adversely impact diabetic eye care more so than concordant comorbidities. The existing literature shows conflicting evidence about the potential negative impact of discordant comorbidities on diabetes management. Using the Veterans Affairs National Patient Care Database, Woodard et al. found those with concordant or discordant conditions were actually more likely than those with no comorbidities to receive good‐quality care for glycemic, blood pressure, and low‐density lipoprotein (LDL) cholesterol control as recommended by the ADA.^27^ However, in another study by Pentakota et al., after accounting for frequency of visits at baseline, the authors found that having concordant comorbidities was associated with similar or better diabetes care, and having discordant comorbidities was associated with decreased diabetes care, as measured by diabetes‐related care measures including guideline‐consistent testing and treatment goals for glycosylated hemoglobin, LDL cholesterol, and diabetes‐related outpatient visits.^10^ For receipt of diabetic eye examinations specifically, there is also conflicting evidence on the role of comorbidity status. Using the Medical Expenditure Panel Survey, An et al. found patients with more than one discordant comorbidity had higher odds of self‐reported dilated eye examination compared to those with diabetes only, and patients with concordant‐only comorbidities had similar odds of eye examinations compared to the diabetes‐only group.^7^ In another retrospective cohort study using healthcare administrative databases based in Canada, the odds of at least one eye examination in a 2‐year period was higher in all groups, diabetes‐concordant only, discordant only, and concordant + discordant, as compared to the no‐comorbidity group.^13^ Neither of these studies accounted for history of eye disease, which is a known predictor of receiving eye examinations.^28^ Another explanation for the conflicting results is that some studies included an adjustment for frequency of visits at baseline as a confounder while others did not. Our study is unique in that we consider baseline healthcare utilization as an effect measure modifier rather than a confounder, based on the evidence of utilization impacting frequency of diabetes preventive care.^15^

The challenges of competing interests from multiple comorbid conditions are likely exacerbated by the presence of discordant conditions.^9^, ^26^, ^29^ Some diabetes‐discordant conditions, for example cancer, may require encounters with other specialists who might not be addressing diabetes altogether. Our findings suggest that the negative impact of comorbid conditions is mitigated by an increased number of outpatient contacts—comorbidities were not associated with lower prevalence of eye examinations among the high healthcare utilizers. The increased healthcare utilization possibly results in increased opportunities for diabetes‐related health monitoring and counseling resulting in improved diabetes preventive care.^9^

Receipt of recommended diabetic eye examinations is likely particularly sensitive, even more so than other metrics of diabetes care, to competing demands as it traditionally requires a separate outpatient visit with an optometrist or ophthalmologist. Furthermore, many vision‐threatening complications of diabetic retinopathy are asymptomatic until the late stages. This study highlights a particular subset of patients with diabetes identifiable on the basis of their comorbid conditions and overall healthcare utilization that would benefit most from increased assistance in obtaining vision‐preserving eye examinations. Assistance could be in the form of new models of eye care delivery, including automated diabetic retinopathy detection using artificial intelligence.^30^ This technology allows diabetic retinopathy screening to occur in primary care physician offices, decreasing the burden of needing to go to a separate eye doctor appointment. Expanding the reach of such an algorithm to locations beyond the healthcare system to commercial pharmacies, for example, may be beneficial by further decreasing barriers to eye care and allowing diabetic retinopathy screening to take place in the community.

The impact of the other covariates on receipt of diabetic eye examinations is in line with previous studies. Younger male patients were less likely to receive eye examinations.^31^, ^32^, ^33^ Prevalence of eye examinations also varied by region.^34^ Finally, those with major eye diseases were also more likely to receive eye care.^28^

There are several limitations to our study. Since this is an observational study, we are unable to demonstrate a causal relationship between comorbidities, infrequent healthcare utilization, and lower prevalence of eye examination. Although some of our findings were statistically significant, the overall effect sizes were small. The MarketScan database is not a nationally representative sample and only represents a subset of the insured population. Administrative claims data are not always accurate for clinical diagnoses, although we are applying previously used and validated algorithms in this study. We excluded patients with fewer than three diabetes diagnosis codes who could not be differentiated between type 1 and type 2 diabetes. Although this was a small group, only 5% of those with diabetes, we could be excluding a group of particularly low healthcare utilizers. We could be underestimating receipt of diabetic eye examinations if they were performed outside of the insurance network and not captured in this database, for example paying cash for an optometry visit. We suspect this is uncommon as most health insurance plans cover diabetic eye examinations. We are also inferring that a diabetic retinopathy examination took place at these ophthalmic visits since these are the codes accepted by the Healthcare Effectiveness Data and Information Set (HEDIS) to indicate the eye examination component of comprehensive diabetes care. However, without access to the clinical record for each patient, we cannot verify to what degree this assumption is true. Finally, due to limitations of the database, we do not have information on patient race, ethnicity, baseline vision impairment, or other disabilities which have been shown to impact receipt of eye examinations.

In summary, having comorbid conditions can negatively impact receipt of recommended biennial or annual eye examinations, but this negative impact appears to be mitigated by higher healthcare utilization. Having concordant or concordant + discordant comorbidity among the low healthcare utilizers and a discordant comorbidity among the medium utilizers, in general, were associated with lower prevalence of eye examinations. Our study highlights populations of patients with diabetes who would benefit from new modes of eye care delivery to decrease the barriers to eye examinations. This study also indicates the importance of all types of healthcare providers, not just those primarily managing diabetes, to be mindful of the ADA recommendations for diabetic eye examinations and to encourage their patients to receive vision‐saving screenings and treatments.

## FUNDING INFORMATION

None.

## CONFLICT OF INTEREST

Cindy X. Cai, Minchul Kim, Elizabeth A. Lundeen, Stephen R. Benoit: None.

## DISCLAIMER

The findings and conclusions in this report are those of the authors and do not necessarily represent the official position of the Centers for Disease Control and Prevention.

## Supporting information


**FIGURE S1** Flow diagram for identification of the longitudinal cohort of patients with type 2 diabetes mellitus in the MarketScan database

**TABLE S1** Key definitions
**TABLE S2** Defining diabetes concordant and discordant comorbidities
**TABLE S3** Sensitivity analyses of the unadjusted and adjusted prevalence ratio of receiving recommended annual eye examination
