# Supplementary figures and images for: Differences in receipt of recommended eye examinations by comorbidity status and healthcare utilization among nonelderly adults with diabetes
Source: J Diabetes. 2022 Oct 26;14(11):749–57. doi: 10.1111/1753-0407.13328 (PMC9705799; doi:10.1111/1753-0407.13328)

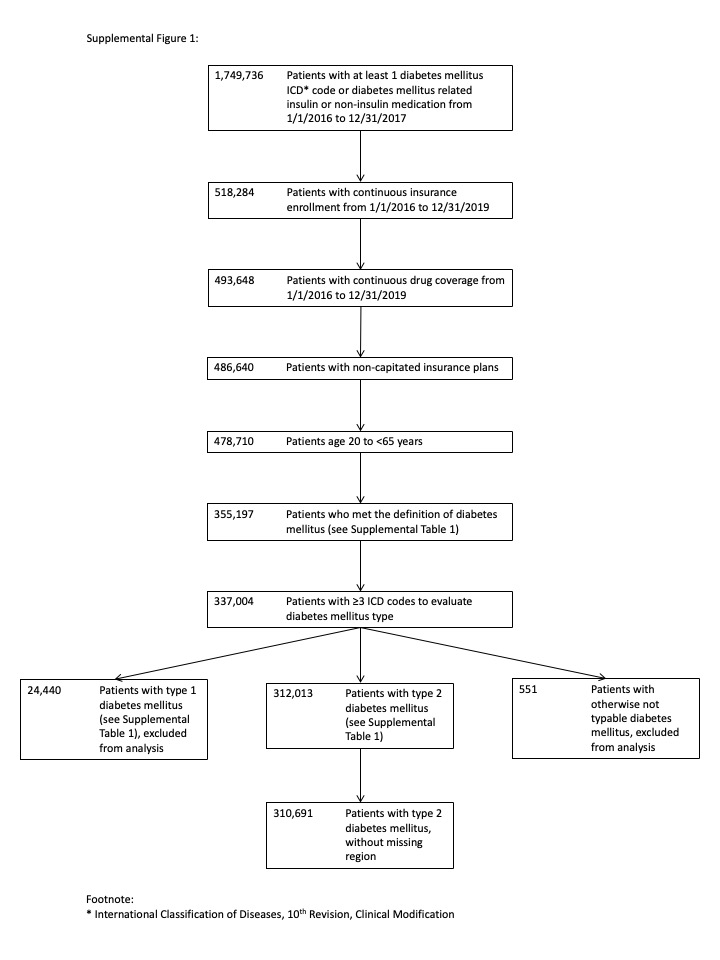

Supplement: Supplementary file 1 — FIGURE S1 Flow diagram for identification of the longitudinal cohort of patients with type 2 diabetes mellitus in the MarketScan database [file JDB-14-749-s001.jpg]
