# Supplementary material for: Differences in receipt of recommended eye examinations by comorbidity status and healthcare utilization among nonelderly adults with diabetes
Source: J Diabetes. 2022 Oct 26;14(11):749–57. doi: 10.1111/1753-0407.13328 (PMC9705799; doi:10.1111/1753-0407.13328)
Supplement: Supplementary file 2 — TABLE S1 Key definitions TABLE S2 Defining diabetes concordant and discordant comorbidities TABLE S3 Sensitivity analyses of the unadjusted and adjusted prevalence ratio of receiving recommended annual eye examination [file JDB-14-749-s002.docx]

Supplemental Table 1: Key definitions

|  | Definition |
| --- | --- |
| Diabetes mellitus | 1) 1 inpatient ICD-10* code for diabetes (E10, E11, E13)  or  2) Any combination of 2 instances of the following:  A) Outpatient ICD-10 diagnosis code for diabetes  B) Filled prescription for insulin or non-insulin diabetes medication  Of note: 2 outpatient codes for diabetes qualify but only if the events occurred on separate days. 2 diabetes medications prescriptions also qualify, however, prescriptions for metformin, thiazolidinediones, or GLP-1 agonists had to be combined with another event to qualify. |
| Type 1 diabetes mellitus | 1) >50% ICD-10 codes were E10 (type 1 diabetes mellitus)  and  2) No prescription for sulfonylurea |
| Type 2 diabetes mellitus | 1) >50% ICD-10 codes were E11 (type 2 diabetes mellitus)  or  2) Not type 1 diabetes and had prescription for non-insulin diabetes medication |
| Non-insulin diabetes medications | Metformin, Sulfonylurea, Thiazolidinediones, Glucagon-like peptide (GLP)-1 agonists, meglitinides, sodium-glucose cotransporter-2 (SGLT-2) inhibitors |
| Eye exam | CPT** code: 67028, 67030, 67031, 67036, 67038, 67039, 67040, 67041, 67042, 67043, 67101, 67105, 67107, 67108, 67110, 67112, 67113, 67121, 67141, 67145, 67208, 67210, 67218, 67220, 67221, 67227, 67228, 92002, 92004, 92012, 92014, 92018, 92019, 92134, 92225, 92226, 92227, 92228, 92230, 92235, 92240, 92250, 92260, 2022F, 2024F, 2026F, 3072F, 2023F, 2025F, 2033F  CPT code: 99201 – 99205, 99212 – 99215, 99241 – 99245, 99341 – 99350 + provider type optometrist or ophthalmologist  HCPCS*** code: S0620, S0621, S0625, S3000  ICD-10 diagnosis code: Z01.00, Z01.01 |
| Major eye disease | Age-Related Macular Degeneration:   - Non-neovascular age-related macular degeneration: H35.31 - Neovascular age-related macular degeneration: H35.32   Glaucoma:   - Primary open angle glaucoma: H40.11, H40.12 - Primary closed angle glaucoma: H40.2 |
| Outpatient Encounters | Place of Service at: Office, Walk-in Retail Health Clinic, Outpatient Hospital-Off Campus, Outpatient Hospital-On Campus, Independent Clinic, Federally Qualified Health Center, Outpatient (NEC) |

* International Classification of Diseases, 10^th^ Revision, Clinical Modification

** Current Procedural Terminology

*** Healthcare Common Procedure Coding System

Supplemental Table 2: Defining diabetes concordant and discordant comorbidities

| Concordant Comorbidity | Discordant Comorbidity |
| --- | --- |
| Hypertension, Hyperlipidemia, Congestive heart failure, Coronary artery disease, Cardiac arrhythmia, Stroke, Chronic kidney disease | Arthritis, Osteoporosis, All types of cancer except for non-melanoma skin cancer, Asthma, Chronic obstructive pulmonary disease, Dementia, Depression, Autism spectrum disorder, Schizophrenia, Liver disease, Human immunodeficiency virus, Substance abuse disorder |

Supplemental Table 3: Sensitivity analyses of the unadjusted and adjusted prevalence ratio of receiving recommended annual eye examination

|  | Received Recommend Eye Exam  N (%) | Unadjusted Prevalence Ratio (95% CI*) | p-value | Adjusted Prevalence Ratio (95% CI) | p-value |
| --- | --- | --- | --- | --- | --- |
| Comorbidity Status |  |  |  |  |  |
| Diabetes only | 1,092 (15.9) | (reference) |  |  |  |
| Concordant only | 29,525 (20.9) | 1.31 (1.24, 1.38) | <0.001 |  |  |
| Discordant only | 773 (16.6) | 1.04 (0.96, 1.13) | 0.359 |  |  |
| Concordant and Discordant | 37,139 (23.6) | 1.48 (1.40, 1.56) | <0.001 |  |  |
| Healthcare Utilization^‡^ |  |  |  |  |  |
| Low (0 to 13) | 14,007 (13.3) | (reference) |  |  |  |
| Medium (14 to 27) | 23,834 (23.0) | 1.72 (1.69, 1.76) | <0.001 |  |  |
| High (28 to 731) | 30,688 (30.2) | 2.27 (2.23, 2.31) | <0.001 |  |  |
| Comorbidity Status Stratified by Healthcare Utilization |  |  |  |  |  |
| Low (0 to 13) |  |  |  |  |  |
| Diabetes only |  |  |  | (reference) |  |
| Concordant only |  |  |  | 0.97 (0.90, 1.04) | 0.407 |
| Discordant only |  |  |  | 0.80 (0.69, 0.94) | 0.006 |
| Concordant and Discordant |  |  |  | 0.86 (0.79, 0.92) | <0.001 |
| Medium (14 to 27) |  |  |  |  |  |
| Diabetes only |  |  |  | (reference) |  |
| Concordant only |  |  |  | 1.03 (0.94, 1.12) | 0.547 |
| Discordant only |  |  |  | 0.74 (0.65, 0.85) | <0.001 |
| Concordant and Discordant |  |  |  | 0.84 (0.77, 0.92) | <0.001 |
| High (28 to 731) |  |  |  |  |  |
| Diabetes only |  |  |  | (reference) |  |
| Concordant only |  |  |  | 1.30 (1.12, 1.50) | <0.001 |
| Discordant only |  |  |  | 0.99 (0.84, 1.17) | 0.908 |
| Concordant and Discordant |  |  |  | 1.15 (0.99, 1.32) | 0.062 |
| Age |  |  |  |  |  |
| 20 to <40 years | 1,222 (10.2) | (reference) |  | (reference) |  |
| 40 to <50 years | 7,945 (15.0) | 1.47 (1.39, 1.55) | <0.001 | 1.33 (1.27, 1.39) | <0.001 |
| 50 to <60 years | 30,636 (21.1) | 2.07 (1.96, 2.19) | <0.001 | 1.68 (1.62, 1.75) | <0.001 |
| 60 to <65 years | 28,726 (28.5) | 2.79 (2.65, 2.95) | <0.001 | 2.11 (2.03, 2.19) | <0.001 |
| Sex |  |  |  |  |  |
| Female | 33,281 (23.3) | (reference) |  | (reference) |  |
| Male | 35,248 (21.0) | 0.90 (0.89, 0.91) | <0.001 | 0.96 (0.95, 0.97) | <0.001 |
| Region |  |  |  |  |  |
| Northeast | 16,245 (27.6) | (reference) |  | (reference) |  |
| North Central | 13,832 (23.4) | 0.85 (0.83, 0.86) | <0.001 | 0.92 (0.90, 0.94) | <0.001 |
| South | 33,089 (20.5) | 0.74 (0.73, 0.76) | <0.001 | 0.81 (0.80, 0.82) | <0.001 |
| West | 5,363 (17.0) | 0.61 (0.60, 0.63) | <0.001 | 0.71 (0.69, 0.73) | <0.001 |
| Major Eye Disease |  |  |  |  |  |
| No | 61,816 (20.5) | (reference) |  | (reference) |  |
| Yes | 6,713 (68.5) | 3.34 (3.29, 3.39) | <0.001 | 2.97 (2.92, 3.02) | <0.001 |

* Confidence Interval

^‡^ Number of outpatient encounters in the baseline 2-year period
